# Supplementary material for: Viral Capsid-Membrane Interactions Propel Non-Brownian Movements of Non-enveloped Reoviruses during Entry
Source: bioRxiv. 2026 Jan 22:2026.01.21.700940. Preprint. [Version 1] doi: 10.64898/2026.01.21.700940 (PMC12871715; doi:10.64898/2026.01.21.700940)
Supplement: Supplement 1 [file NIHPP2026.01.21.700940v1-supplement-1.pdf]

bioRxiv preprint doi: https://doi.org/10.64898/2026.01.21.700940; this version posted January 22, 2026. The copyright holder for this preprint (which was not certified by peer review) is the author/funder, who has granted bioRxiv a license to display the preprint in perpetuity. It is made available under aCC-BY 4.0 International license.

Supplementary Information for

Viral Capsid-Membrane Interactions Propel Non-Brownian Movements of Non-enveloped Reoviruses during Entry

Mengchi Jiao,[1,†] Gregory R. Cantrall,[2] Yanqi Yu,[1] Anthony J. Snyder,[3] Steven M. Abel,[2,*] Pranav Danthi,[3,*] Yan Yu[4,*]

[1]Department of Chemistry, Indiana University, Bloomington, IN 47405-7102

[2]Department of Chemical and Biomolecular Engineering, University of Tennessee, Knoxville, TN 37996

[3]Department of Biology, Indiana University, Bloomington, IN 47405-7102

[4]Department of Chemistry, Department of Biomedical Engineering, Washington University in St. Louis, St. Louis, MO 63130

[†] Current address: Division of Immunobiology, Cincinnati Children's Hospital Medical Center, Cincinnati, OH 45229

[*]Corresponding authors: abel@utk.edu; pdanthi@iu.edu; yuy1@wustl.edu

ORCID: Yan Yu (0000-0001-6496-5045), Pranav Danthi (0000-0001-6199-6022), Mengchi Jiao (0000-0001-5958-0706), Yanqi Yu (0000-0002-6969-3752), Anthony J. Snyder (0000-0002-2551-9461), Steven M. Abel (0000-0003-0491-8647), Gregory R. Cantrall (0000-0003-4138-4971)

Contents

Supplementary figures S1-S8

bioRxiv preprint doi: https://doi.org/10.64898/2026.01.21.700940; this version posted January 22, 2026. The copyright holder for this preprint (which was not certified by peer review) is the author/funder, who has granted bioRxiv a license to display the preprint in perpetuity. It is made available under aCC-BY 4.0 International license.

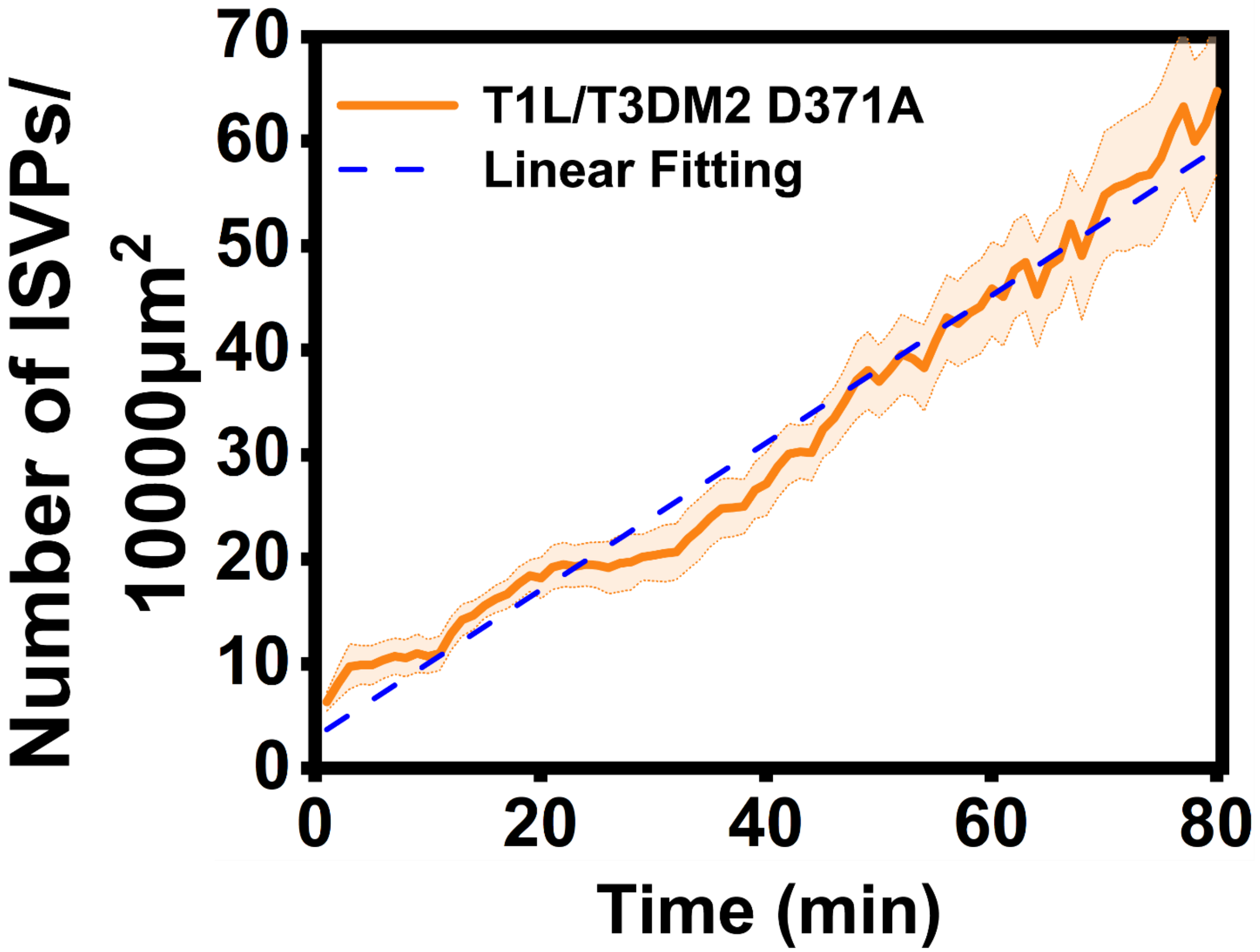

Figure S1. Recruitment of mutant reovirus ISVPs on the planar-supported lipid bilayer. Averaged line plots showing the adsorption of 10 pM T1L/T3DM2 D371A ISVPs on supported lipid bilayer along time. Error bar represents the standard error of the mean (S.E.M).

bioRxiv preprint doi: https://doi.org/10.64898/2026.01.21.700940; this version posted January 22, 2026. The copyright holder for this preprint (which was not certified by peer review) is the author/funder, who has granted bioRxiv a license to display the preprint in perpetuity. It is made available under aCC-BY 4.0 International license.

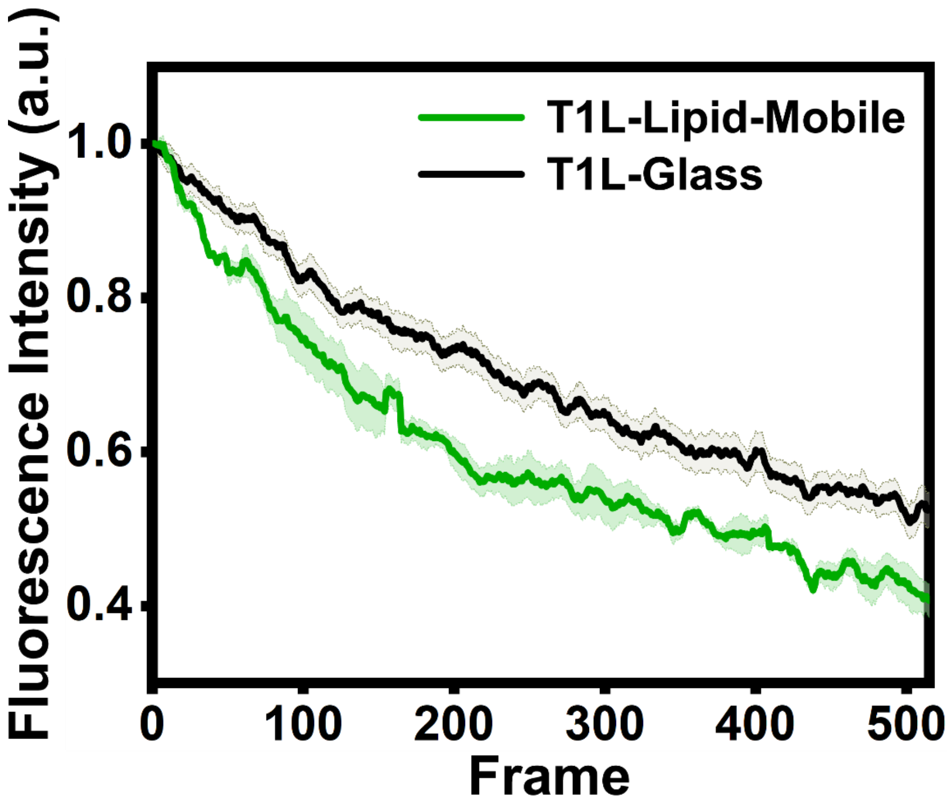

Figure S2. **Capsid uncoating of T1L ISVPs during the interaction with lipids**. Averaged line plots showing the fluorescence intensity of T1L ISVP on different surfaces over time. The error bar represents the standard error of the mean (S.E.M.).

bioRxiv preprint doi: https://doi.org/10.64898/2026.01.21.700940; this version posted January 22, 2026. The copyright holder for this preprint (which was not certified by peer review) is the author/funder, who has granted bioRxiv a license to display the preprint in perpetuity. It is made available under aCC-BY 4.0 International license.

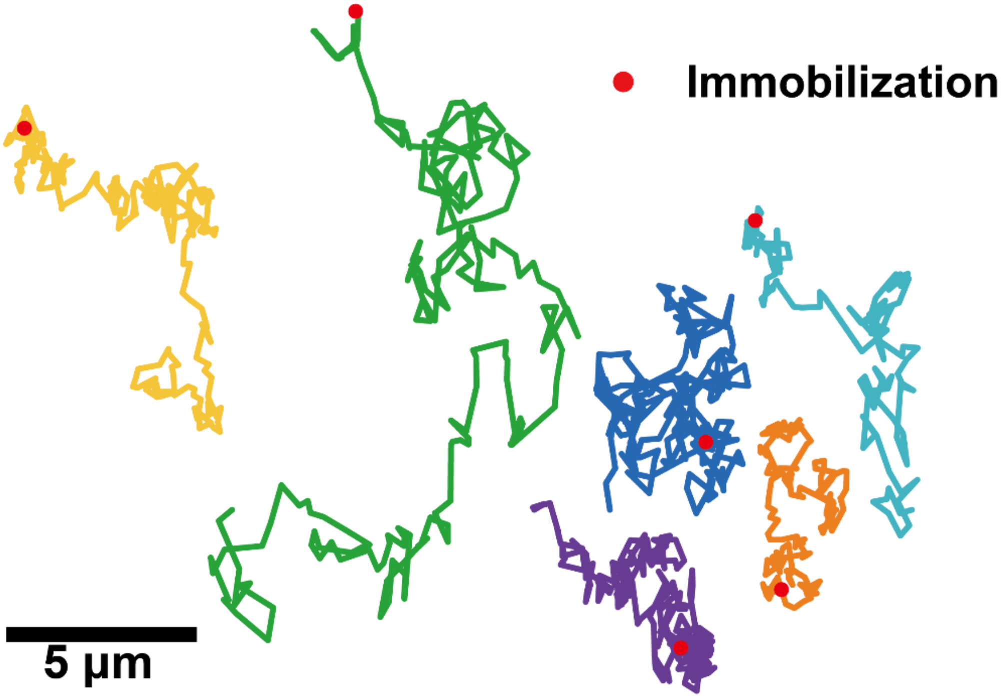

Figure S3. **Complete immobilization of T1L/T3DM2 during imaging**. Line plots showing the trajectories of T1L/T3DM2 that were completely immobilized on the membrane after interactions with lipids

bioRxiv preprint doi: https://doi.org/10.64898/2026.01.21.700940; this version posted January 22, 2026. The copyright holder for this preprint (which was not certified by peer review) is the author/funder, who has granted bioRxiv a license to display the preprint in perpetuity. It is made available under aCC-BY 4.0 International license.

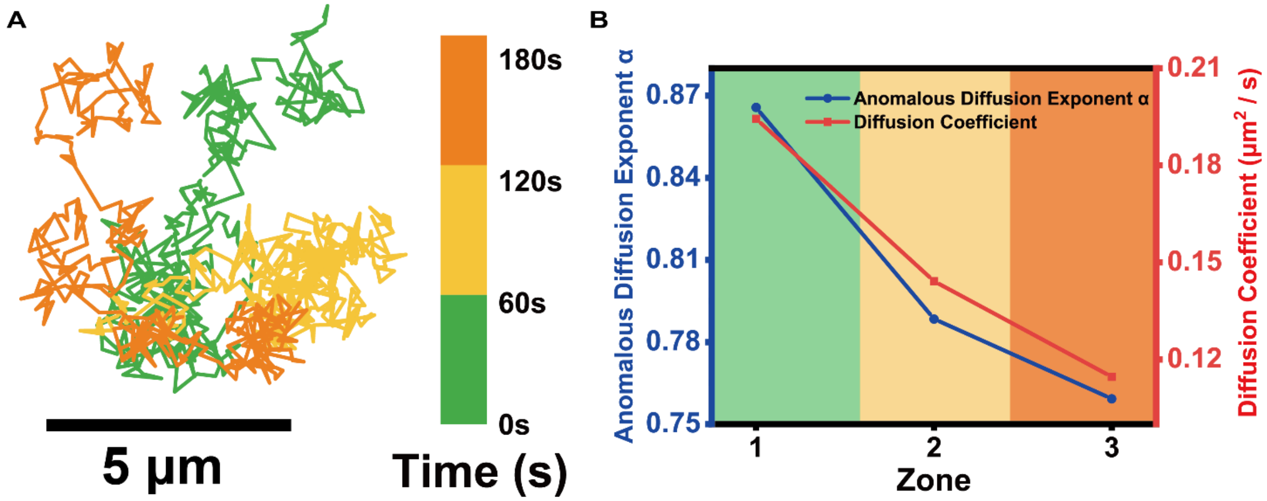

Figure S4. **Confinement of T1L/T3DM2 ISVP increased over time on the planar-supported lipid bilayer**. (A) Line plot showing a trajectory of a T1L/T3DM2 ISVP on the planar-supported lipid bilayer color-coded with time. (B) Line plots showing the corresponding anomalous diffusion exponent and diffusion coefficient change along with time from the trajectory shown in (A).

bioRxiv preprint doi: https://doi.org/10.64898/2026.01.21.700940; this version posted January 22, 2026. The copyright holder for this preprint (which was not certified by peer review) is the author/funder, who has granted bioRxiv a license to display the preprint in perpetuity. It is made available under aCC-BY 4.0 International license.

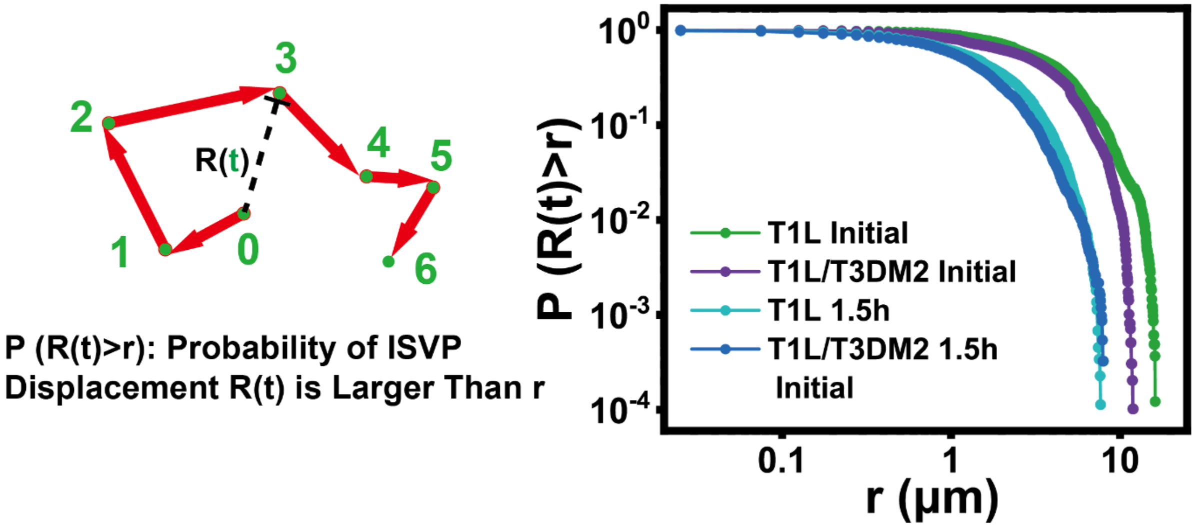

Figure S5. **Probability of ISVP displacement**. Schematic illustration of the displacement of ISVP R(t) in a trajectory (left) and log-log line plots (right) showing the probability of locating an ISVP outside of r distance away from the origin. T1L initial, n=8155; T1L/T3DM2 initial, n=9796; T1L after 1.5h, n=8786; T1L/T3DM2 after 1.5h, n=9196

bioRxiv preprint doi: https://doi.org/10.64898/2026.01.21.700940; this version posted January 22, 2026. The copyright holder for this preprint (which was not certified by peer review) is the author/funder, who has granted bioRxiv a license to display the preprint in perpetuity. It is made available under aCC-BY 4.0 International license.

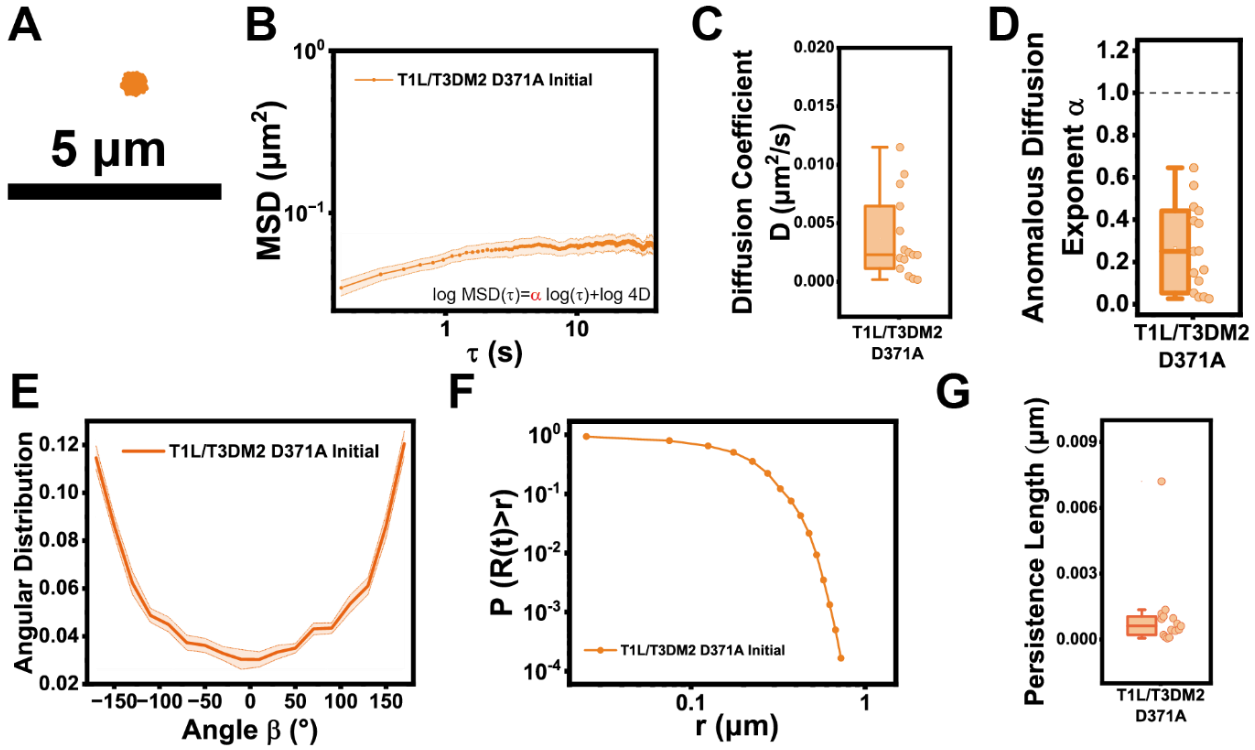

Figure. S6 Translational diffusion of mutant ISVPs on the planar-supported lipid bilayer. (A) Representative line plots showing the trajectories of T1L/T3DM2 D371A ISVPs on the supported lipid bilayer. Scale bar, 5 μm. (B) Averaged log-log line plots showing the mean square displacement of ISVP trajectories. T1L/T3DM2 D371A initial (n=15). Error bar represents the S.E.M. (C and D) Statistical analysis of diffusion coefficient (C) and anomalous diffusion exponent (D) of trajectories from T1L/T3DM2 D371A initial (n=15). (E) Averaged line plot showing the angular distribution of turning angle in trajectories of T1L/T3DM2 D371A (n=15) ISVPs right after the recruitment of ISVPs on the planar-supported lipid bilayer. Error bar represents the standard error of the mean (S.E.M). (F) log-log line plots showing the probability of locating an ISVP outside of r distance away from the origin. T1L/T3DM2 D371A initial, n=5995. (G) Box and scatter plot of persistence length of trajectories from T1L/T3DM2 D371A initial (n=15). Each boxplot indicates the interquartile range from 25% to 75% of the corresponding data set. The mean and median are demonstrated as the square and the horizontal line, respectively. Statistical significance is highlighted by p values.

bioRxiv preprint doi: https://doi.org/10.64898/2026.01.21.700940; this version posted January 22, 2026. The copyright holder for this preprint (which was not certified by peer review) is the author/funder, who has granted bioRxiv a license to display the preprint in perpetuity. It is made available under aCC-BY 4.0 International license.

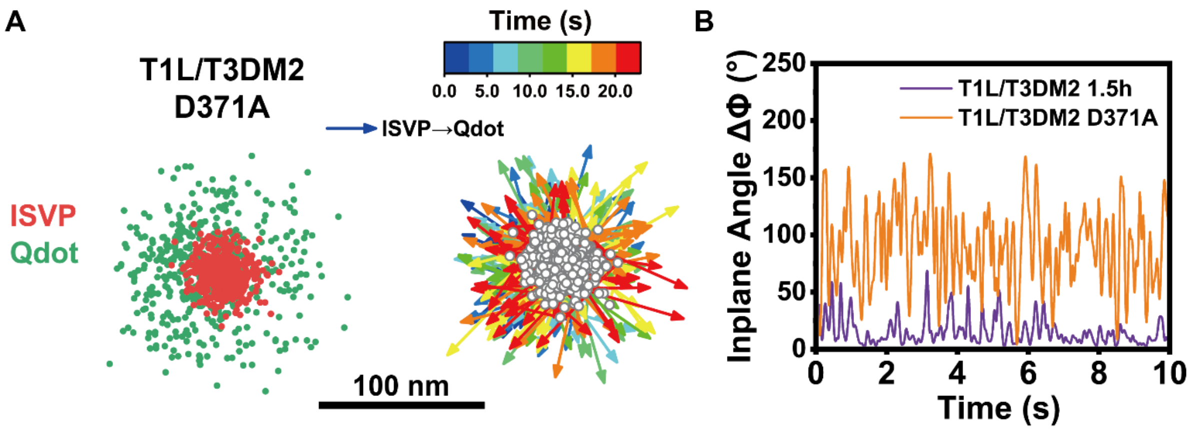

Figure S7. Rotational tracking of T1L/T3DM2 D371A ISVPs on the planar-supported lipid bilayer. (A) Representative rotational trajectories of T1L/T3DM2 D371A ISVP on the planar-supported lipid bilayer. Colormap encodes temporal information. (B) Line plots showing the change of in-plane angle in the trajectories shown in (A) and Fig. 5C.

bioRxiv preprint doi: https://doi.org/10.64898/2026.01.21.700940; this version posted January 22, 2026. The copyright holder for this preprint (which was not certified by peer review) is the author/funder, who has granted bioRxiv a license to display the preprint in perpetuity. It is made available under aCC-BY 4.0 International license.

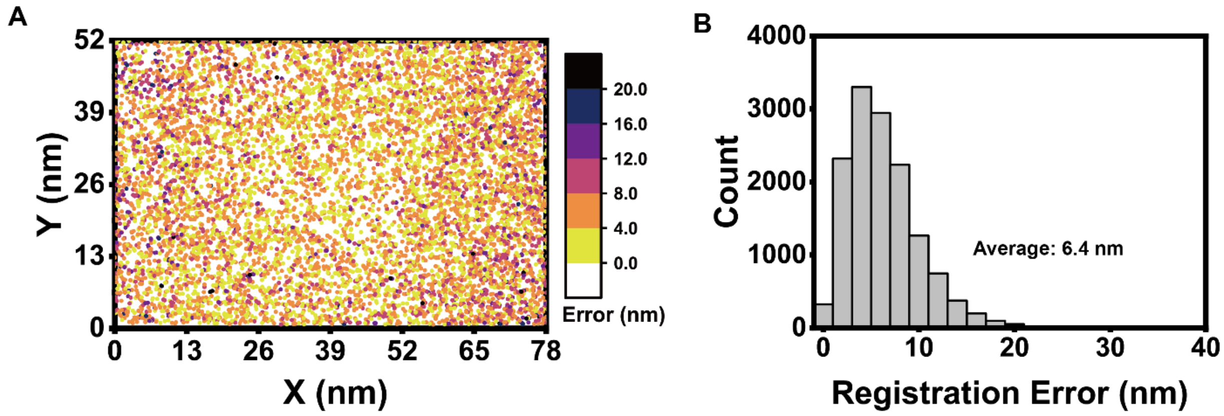

Figure. S8 Registration performance of rotational Tracking. (A) A local registration error map showing the registration error of a set of fiducial markers after the local weighted color mapping. Colormap encodes the magnitude of the local mapping error at each location. (B) Histogram showing registration errors after a local weighted mapping was applied to a set of fiducial markers.
